# Dynamic changes in gene-to-gene regulatory networks in response to SARS-CoV-2 infection

**DOI:** 10.1038/s41598-021-90556-1

**Published:** 2021-05-27

**Authors:** Yoshihisa Tanaka, Kako Higashihara, Mai Adachi Nakazawa, Fumiyoshi Yamashita, Yoshinori Tamada, Yasushi Okuno

**Affiliations:** 1 Graduate School of Pharmaceutical Sciences, Kyoto University, Kyoto, 606-8507 Japan; 2 Biomedical Computational Intelligence Unit, HPC- and AI-driven Drug Development Platform Division, RIKEN Center for Computational Science, Kobe, 650-0047 Japan; 3 Graduate School of Medicine, Kyoto University, Kyoto, 606-8507 Japan; 4 Present Address: Innovation Center for Health Promotion, Hirosaki University, Hirosaki, 036-8562 Japan

**Keywords:** Gene regulatory networks, Computational models

## Abstract

The current pandemic of SARS-CoV-2 has caused extensive damage to society. The characterization of SARS-CoV-2 profiles has been addressed by researchers globally with the aim of resolving this disruptive crisis. This investigation process is indispensable to understand how SARS-CoV-2 behaves in human host cells. However, little is known about the systematic molecular mechanisms involved in the effects of SARS-CoV-2 infection on human host cells. Here, we present gene-to-gene regulatory networks in response to SARS-CoV-2 using a Bayesian network. We examined the dynamic changes in the SARS-CoV-2-purturbated networks established by our proposed framework for gene network analysis, thus revealing that interferon signaling gradually switched to the subsequent inflammatory cytokine signaling cascades. Furthermore, we succeeded in capturing a COVID-19 patient-specific network in which transduction of these signals was concurrently induced. This enabled us to explore the local regulatory systems influenced by SARS-CoV-2 in host cells more precisely at an individual level. Our panel of network analyses has provided new insights into SARS-CoV-2 research from the perspective of cellular systems.

## Introduction

The newly emerging coronavirus, severe acute respiratory syndrome coronavirus 2 (SARS-CoV-2), has spread rapidly worldwide^1,2^, with more than 24,000,000 cases of coronavirus disease 2019 (COVID-19) and 830,000 deaths as of August 28, 2020^3^. This pandemic outbreak has drastically changed our society and has compelled us to be vigilant of the continuous risk of SARS-CoV-2 infection^4^. To overcome this dire situation, the development of novel drugs or vaccines continues to be an urgent global challenge. During the therapeutic development process, the elucidation of cellular mechanisms is essential for the discovery of potential targets; the fundamental question to be solved is how the SARS-CoV-2 influences host cells and causes COVID-19 at the molecular level. However, the cellular mechanisms underlying COVID-19 are poorly understood.

High-throughput technologies have contributed to the acquisition of a large amount of “omics” data, which has provided comprehensive information on cellular systems. These technologies have also been utilized during the current research into SARS-CoV-2. Several reports have provided various clues to understanding the global cellular signatures in response to SARS-CoV-2 infection at both the proteome and transcriptome levels^5–7^. Recently, network-based approaches have attracted great interest in the use of emerging omics data for drug discovery and systems biological analysis in the current field of SARS-CoV-2 research^8–13^. Their major approaches combine publicly available sources, including knowledge of the already established pathways and drugs with these omics data to reconstruct molecular networks. However, these networks do not sufficiently represent a real cellular system for the following two main reasons: 1) public data consist of heterogeneous knowledge that has been accumulated throughout longstanding biological research; and 2) previous studies use mixed networks that combine data from various samples, but cannot reflect an individual cell-/patient-specific network.

**Figure 1:**
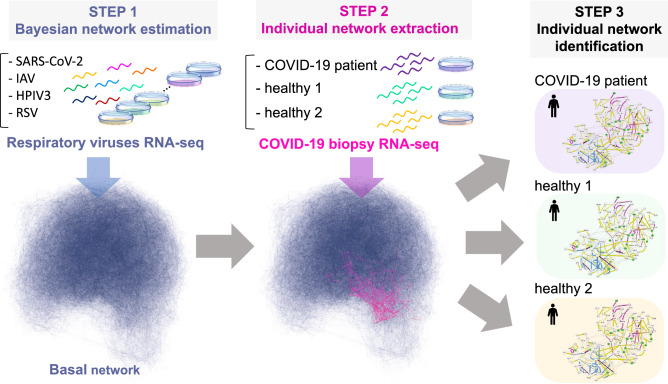
Illustration of overview. The hairball (blue) is the basal network consisting of 127,126 edges and 15,258 nodes established using the respiratory viruses RNA-seq including SARS-CoV-2. The highlighted-network (magenta) in the basal network represents the COVID-19-perturbed network extracted by using the biopsy RNA-seq.

To address these problems, we recently developed a method to extract a core sample-specific network from a massive gene network generated from a Bayesian network^14^. Gene regulatory network estimation has been developed as a prospective method to model the cellular system using omics data^15–19^. Although the Bayesian network-based approach can infer the cause-and-effect relationships between genes with transcriptome data, the key issue is the extraction of biologically significant information from a huge, complicated network, which is often sarcastically referred to as a hairball^20^. Our unique framework consists of the following three steps: 1) estimation of a global gene network; 2) extraction of context-specific core networks based on differences in molecular systems from the global network; and 3) identification of a sample or patient-specific network (Fig. 1). The prominent advantage is that it enables us to identify putative context-specific or sample-specific potential sets of edges in the form of a network, that is, gene-to-gene relationships with directions as well as nodes.

In this study, by using our developed framework for gene network analysis, we have presented the core host cellular systems involved in SARS-CoV-2 over several *in vitro* experiments, including different viral loads, cell lines, and respiratory viruses. No studies have been performed on the computational data-driven gene regulatory network approach for SARS-CoV-2. We characterized interferon signaling and subsequent inflammatory signaling cascades as significantly changed networks in human host cells, which represent the innate antiviral immune system in response to SARS-CoV-2 infection. Additionally, recent studies have reported that patients with COVID-19 exhibit various clinical outcomes depending on each patient, and that a certain proportion of patients will experience a severe disease^21–23^. Therefore, it is much more important to reveal the cellular mechanisms causing these clinical symptoms at the individual level. To this end, we have further identified the gene networks specifically for patients with COVID-19. We believe that our landscape of gene networks is beneficial for understanding the mechanisms by which cellular systems respond to SARS-CoV-2 and to further drug development.

## Results

### Estimation of the basal gene network in the involvement of respiratory virus infection using a Bayesian network

We first characterized a global gene network (hereafter referred to as the *basal network*) using a Bayesian network (see Methods) with a transcriptome dataset involved in the engagement of respiratory virus infection, including SARS-CoV-2, in several human cell lines^7^ (Supplementary Table S1). Since the outstanding characteristic of our approach is to capture sample-specific signatures from the basal network, it is preferable that various reactions are included to model complex gene regulatory systems^14^. To determine the basal network structure, we performed a network estimation using the neighbor node sampling and repeat algorithm^24^, and screened the best algorithm parameters for the target dataset, as described in our previous study^14^. Briefly, the network estimation was run three times independently, and the subsequent concordance test was performed to ensure the robustness and stability of the estimated basal network. We confirmed that the iteration number $T=500,000$ satisfied less than 5% error (Error=4.0% for $T=500,000$; error=5.3% for $T=300,000$). The final basal network comprised 127,126 edges and 15,258 nodes, with a threshold of 0.05 and an average degree of 16.7. We used this final basal network for subsequent analyses.

**Figure 2:**
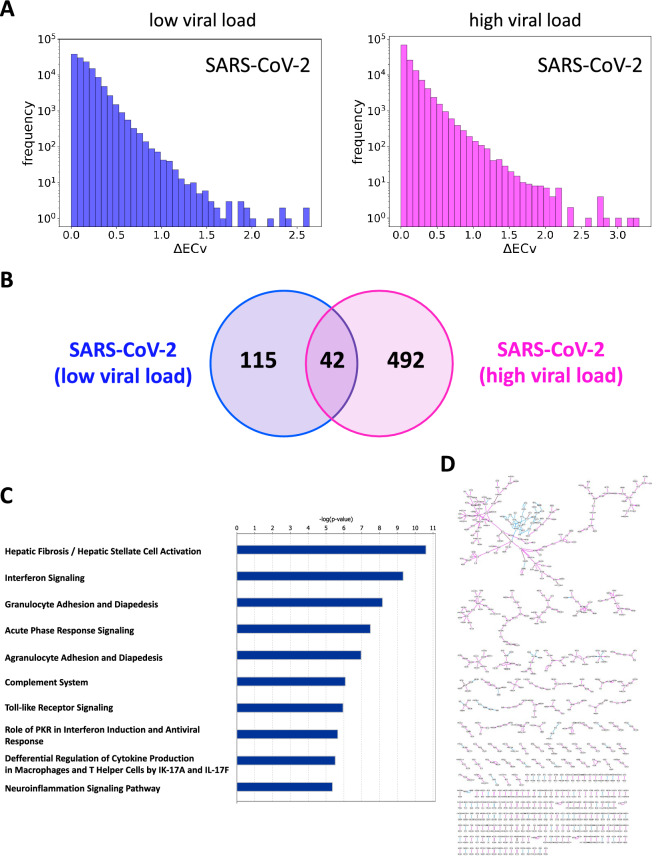
Dynamics of the SARS-CoV-2-perturbed network for different viral loads in host cells. (**A**) The histograms of $\Delta$ECv for different SARS-CoV-2 viral loads; a low MOI of 0.2 (blue) and a high MOI of 2 (magenta). The X-axis corresponds to the threshold for each $\Delta$ECv. The Y-axis shows the number of edges on a log scale. (**B**) The Venn diagram represents the numbers of differentially regulated edges (DREs) for two SARS-CoV-2 viral loads (blue: low MOI, magenta: high MOI) with a threshold of 1.0 for $\Delta$ECv in Fig. 2A. (**C**) The top 10 terms of canonical pathway analysis for the genes comprising a union set of $\Delta$ECv-extracted DREs in the Venn diagram analysis (Fig. 2B). (**D**) The whole illustration for the subnetwork fragments of various sizes is shown. Image of how the $\Delta$ECv-extracted DREs mutually connected and generated the subnetworks.

### Dynamics of host cellular network profiles at different viral loads of SARS-CoV-2

To examine the transition of host cellular system dynamics during the increase of SARS-CoV-2 viral loads, we characterized the networks perturbed by SARS-CoV-2 with two viral loads, namely a low multiplicity of infection (MOI) of 0.2 and a high MOI of 2 in A549 cells. We expected that cells exposed to different viral loads would present a unique cellular system, and that our approach could capture the fluctuation of system dynamics in whole cellular systems. To obtain differential core gene networks for each viral load, we followed multiple steps using an edge quantification technique, called the *edge contribution value* (ECv), established in our previous study^14^. We first calculated $\Delta$ECvs following Eq. (2), where *S* = SARS-CoV-2 infected and *T* = mock samples for each MOI condition (see Methods). The distributions of $\Delta$ECv showed that the innate cellular system was extensively more perturbed in the cells exposed to the high MOI than those exposed to low MOI (Fig. 2A). We next set a threshold of 1 for $\Delta$ECv and obtained *differentially regulated edges* (DREs) from the basal network. The Venn diagram analysis for the $\Delta$ECv-extracted DREs showed that the number of DREs in the high MOI was larger than that in the low MOI (Fig. 2B). Interestingly, the number of shared DREs between high- and low- MOIs was 42, which was only 6% of the total number of DREs in both conditions, thereby indicating that the underlying regulatory system between them was not similar. To confirm the biological involvement of the DREs, we performed canonical pathway analysis for the genes contained in the $\Delta$ECv-obtained DREs, and showed that these genes were associated with some cellular antiviral systems (Fig. 2C). These results support that the components of the DREs are biologically relevant to viral infection.

**Figure 3:**
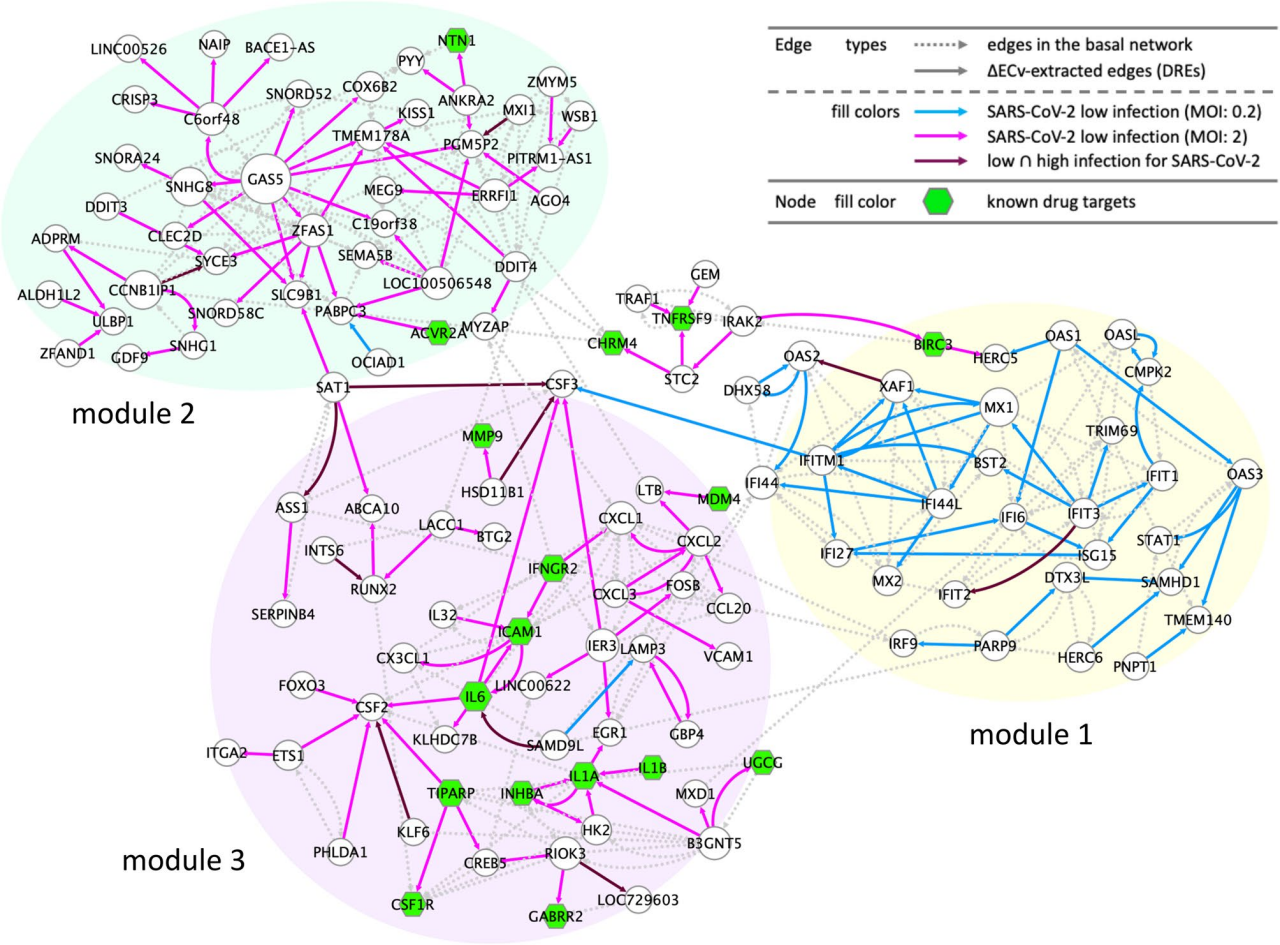
The SARS-CoV-2-perturbed network. The SARS-CoV-2-perturbed host cellular network in response to different viral loads in A549 cells (the SARS-CoV-2-perturbed network). The network comprises 130 nodes and 305 edges (including 155 basal edges). The colored solid edges represent the SARS-CoV-2-perturbed DREs; high MOI of 2 (magenta), low MOI of 0.2 (blue), and high MOI $\cap$ low MOI (purple). Dotted edges represent basal edges (gray). The nodes (green) represent the known drug target genes (Supplementary Table S2). The node size represents the extent of outdegree.

To gain a greater insight into the profiles of the DREs from the perspective of network topology, we next generated networks using a set of all the DREs in the Venn diagram (Fig. 2B). These DREs connected mutually and, in turn, generated subnetwork fragments of various sizes (Fig. 2D). We reasoned that if these fragments had biological significance, these features should be reflected as modular, as biologically close functions in cellular systems link together and shape modules^25^. Hence, small-sized fragments were likely to be less informative, and we focused on the largest connected component among the various fragments. The largest connected component was extracted and the basal edges were additionally mapped on this network, which established the SARS-CoV-2-perturbed network with 130 nodes and 305 edges (Fig. 3). We found that this network clearly consisted of three modules linked to each other. One module (module 1, yellow-marked region) was mainly composed of a set of DREs under low-MOI conditions, and its constituent elements were interferon (IFN)-stimulated genes (ISGs), namely IFIs, MXs, OASs, TRIMs, IFTMs, IRFs, and STATs. These highly orchestrated webs of various ISGs are induced by transductions of both IFN signaling and subsequent JAK/STAT signaling^26^. This evidence strongly suggests that module 1 represents the consequences of activation of both these signaling pathways by the acute antiviral response. Contrary to module 1, the other two modules (module 2, green-marked region; and module 3, purple-marked region) are mainly shaped by a set of DREs in the high-MOI condition. Modules 2 and 3 were found to comprise fewer IFN-related genes. While module 2 appeared to be a GAS5-centralized module, module 3 was composed of chemokines (CXCL1, CXCL2, CXCL3, CX3CL1, and CCL20), interleukins (IL6, IL1A, IL1B, and IL32), and colony-stimulating factors (CSF2 and CSF3), which have been implicated in inflammatory-related cytokine signaling followed by the acute activation of IFN and JAK/STAT signaling represented in module 1. In particular, the cluster of modules 1 and 3 likely represents the transition of the gene regulatory system in response to SARS-CoV-2 infection. Specifically, the cellular system perturbed by SARS-CoV-2 gradually switched to inflammatory signaling (module 3) via IFN and JAK/STAT signaling (module 1) as the viral load increased. This was consistent with the clinical observations of COVID-19, and may thus partially explain the process of cytokine storm syndromes, which is a severe clinical feature of COVID-19^21,23^. We also performed the same analyses among the four respiratory viruses and found that module 3 was exclusive for SARS-CoV-2 (Supplementary Fig. S1). Collectively, we identified the SARS-CoV-2-perturbed network and its three modules, which reflected distinctive host cellular functions in response to SARS-CoV-2 infection.

### Characterization of the SARS-CoV-2-perturbed network at the individual sample level

**Figure 4:**
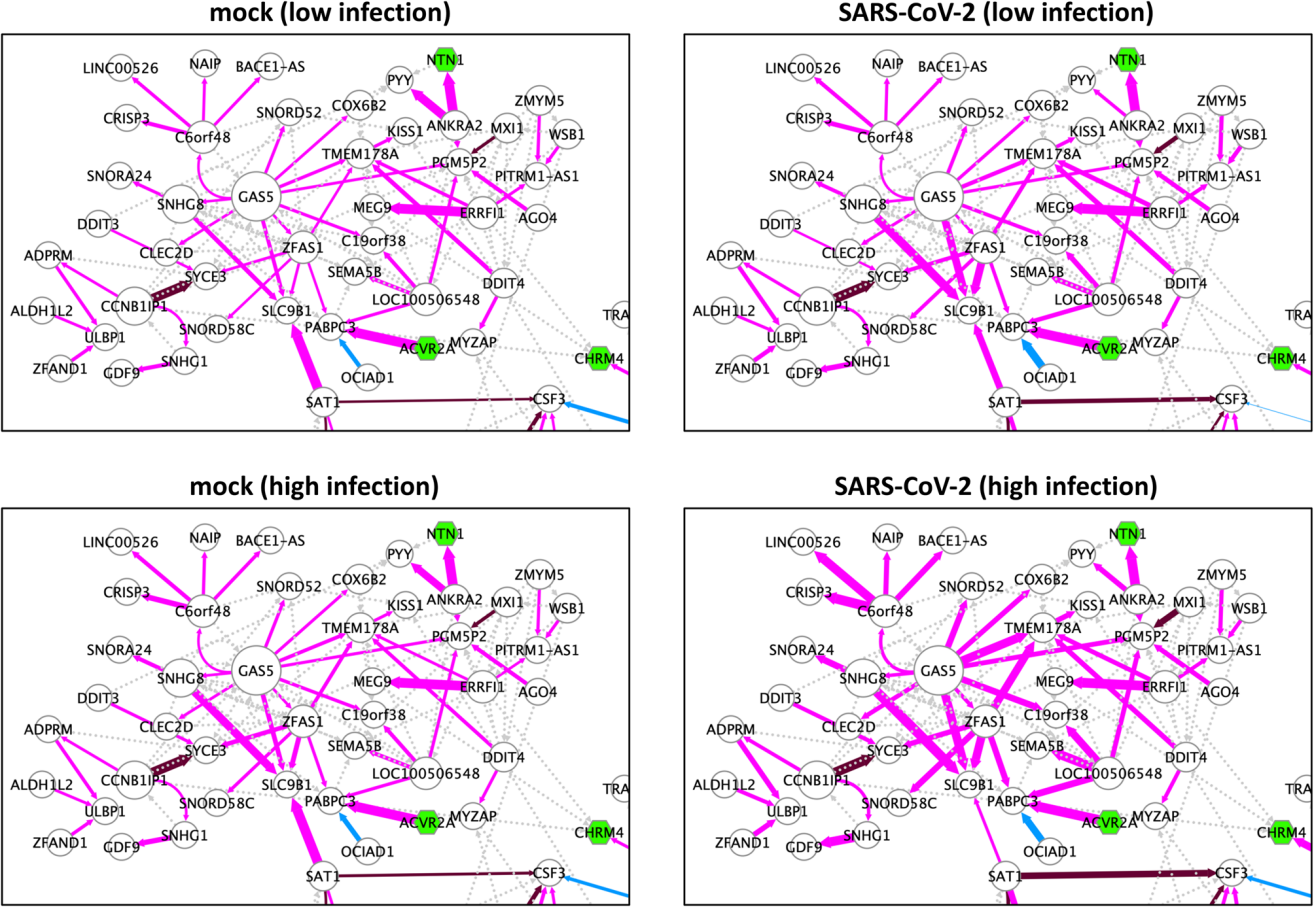
Sample-specific individual networks around the GAS5-centralized module. The GAS5-centralized module (module 3) in the SARS-CoV-2-perturbed network (presented in Fig. 3) is displayed for four representative samples from each group (mock for SARS-CoV-2-infection (low MOI: 0.2), SARS-CoV-2-infected (low MOI: 0.2), mock for SARS-CoV-2-infection (high MOI: 2), and SARS-CoV-2-infected (high MOI: 2)). RCs are represented as edge sizes to show individual differences. The node size represents the extent of outdegree.

Next, we determined how the signaling represented in the SARS-CoV-2-perturbed network (Fig. 3) changed across the samples. To this end, we developed a novel quantitative method, called *relative contribution* (RC), to measure the edge contribution at an individual level. The mathematical definition of RC is described in the Methods section. Within a set of pairwise parent-child relations for a certain child, the RC captures how parent genes influence a child gene in response to the pairwise parent’s mRNA expression, and it can therefore reveal local regulatory changes in response to SARS-CoV-2 infection at an individual sample level. To characterize the individual networks, we calculated RCs for 12 samples within four groups (mock $\times$ 3 for SARS-CoV-2-infected (MOI: 0.2), SARS-CoV-2-infected $\times$ 3 (MOI: 0.2), mock $\times$ 3 for SARS-CoV-2-infected (MOI: 2), SARS-CoV-2-infected $\times$ 3 (MOI: 2)) involved in the network generation process, as shown in Fig. 3. Since we confirmed that the RC profiles exhibit almost the same between the replicates, we selected four representative samples from each group. By representing RCs as the sizes of edge widths, we depicted these four sample-specific individual networks (Supplementary Animation 1), and found that the vicinity of the GAS5-centralized module (module 2) drastically changed at an RC level (Fig. 4). Interestingly, this module included GAS5, SNHG8, ZFAS1, SNORD52, SNORD58C, SNORA24, and LOC100506548, which encode non-coding RNA (ncRNA) genes. Given that GAS5 appears to function as a hub gene, these results suggest that the genes downstream of GAS5 are regulated by different cellular systems in the mock and SARS-CoV-2 infections at a local system level. In particular, GAS5, ZFAS1, and SNHG8 were found to be dominant for SLC9B1 in SARS-CoV-2-infected samples compared with the mock samples, suggesting that the regulatory system used was significantly different between them (Fig. 4). GAS5 is a single-stranded lncRNA, and one study demonstrated that the mRNA expression of GAS5 was elevated in response to hepatitis C virus infection and that GAS5 impaired virus replication by the interaction between truncated-GAS5 and HCV NS3 protein in human cells^27^. Combined with this evidence, our results suggest the possibility that this module 2 related to ncRNA may play a novel clear role in SARS-CoV-2 infection.

Conversely, of the four individual networks, the two networks for mocks exhibited no significant change in RC (Fig. 4 and Supplementary Animation 1). This is consistent with the prerequisite experimental design, as the mock samples are supposed to exhibit the same behavior, which further supports the validity of our method. Moreover, the RC-highlighted edges displaying little to no changes showed that their local regulatory system, presented as a set of pairwise parent-child relationships for one child, did not change between the individual samples. Collectively, our data demonstrate that we can capture the local system differences in network signaling at an individual level.

### Identification of specific individual networks in patients with COVID-19

**Figure 5:**
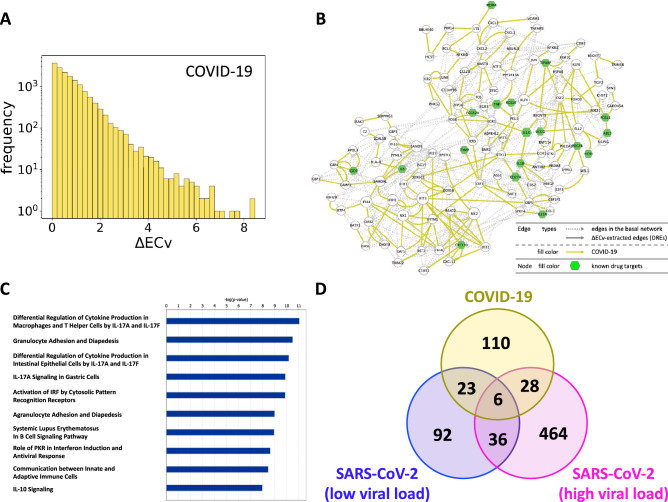
The COVID-19-perturbed network analysis. (**A**) The histograms of $\Delta$ECv for the biopsy dataset. The X-axis corresponds to the threshold for the $\Delta$ECv. The Y-axis stands for the number of edges with log scale. (**B**) The COVID-19-perturbed network is shown. The network is composed of 127 nodes and 412 edges (including 245 basal edges). The colored solid edges represent DREs perturbed by COVID-19 (yellow). The dotted edges represent the basal edges (gray). The nodes (green) represent the known drug target genes (Supplementary Table S2). The node size represents the extent of outdegree. (**C**) The top 10 terms of canonical pathway analysis for the genes in the COVID-19-perturbed network. (**D**) The Venn diagram shows the numbers of the $\Delta$ECv-extracted DREs ($\Delta$ECv threshold 2.3) induced by COVID-19 perturbation for the biopsy dataset (yellow) overlapped with the two DREs through the Venn diagram analysis in Fig. 2B.

Finally, we aimed to establish COVID-19 individual networks with a human biopsy dataset (healthy: two samples; COVID-19-positive: two samples) on the basis of the estimated basal network model. We expect that the *in vivo* biopsy dataset would provide a more clinically relevant perspective compared with the *in vitro* experiments. Usually, network estimation is impossible with such a small number of samples due to the difficulty in acquisition of a robust network structure, yet our approach using the basal network model was capable of generating a context-specific network, even with a few samples of a different dataset (Fig. 1). By using the *B*-spline regression model of the Bayesian network acquired by the estimation of the basal network, we first computed the ECv for the preprocessed biopsy dataset, despite the absence of some genes compared with the dataset used for the basal network estimation. To obtain DREs, we calculated $\Delta$ECv between healthy (regarded as control) and COVID-19-positive samples according to Eq. (2), where *S* = healthy ($|S|=2$) and *T* = COVID-19-positive ($|T|=2$) (see Methods). The $\Delta$ECvs were distributed over a broad range, and 4,242 DREs were observed at a threshold of 1 for $\Delta$ECv (Fig. 5A). To extract more reliable DREs induced by COVID-19, we set a threshold of 2.3, corresponding approximately to $\log _2$FC where FC=5, which resulted in 638 DREs. These DREs were mapped as networks and the largest connected component (167 DREs) was depicted with inclusion of the basal edges, generating the COVID-19-perturbed network, which comprised 127 nodes and 412 edges (Fig. 5B). This network is supposedly a representation of the distinctive cellular system in patients with COVID-19. The pathway analysis of genes contained in this network showed that they were involved in the immune and inflammatory response (Fig. 5C), thereby supporting the consistency of our established network with biological observations in COVID-19.

To determine the signatures of the acquired DREs in the COVID-19-perturbed network, we measured the ECv similarity for a set of the 167 DREs across the other experimental samples. This result showed that the ECv profiles in COVID-19 were similar to the sample with HPIV3 rather than SARS-CoV-2 in the *in vitro* experiments (Supplementary Fig. S2A), thus suggesting that there is a physiological gap between *in vitro* and *in vivo*. We further explored the extent to which the 167 COVID-19-related DREs overlapped with the Venn diagram illustrated in Fig. 2B. We observed that a moderate number of DREs were shared by the cell models of SARS-CoV-2 perturbation (Fig. 5D), then these overlapped edges were mapped onto the COVID-19-perturbed network (Supplementary Fig. S2B). Unlike the network observations shown in Fig. 3, we found that both the ISG-related webs (module 1) and subsequent cytokine signaling (module 3) involved in inflammatory cascades were concurrently present in the COVID-19-perturbed network, indicating that these two modules continued to be mutually activated in COVID-19.

**Figure 6:**
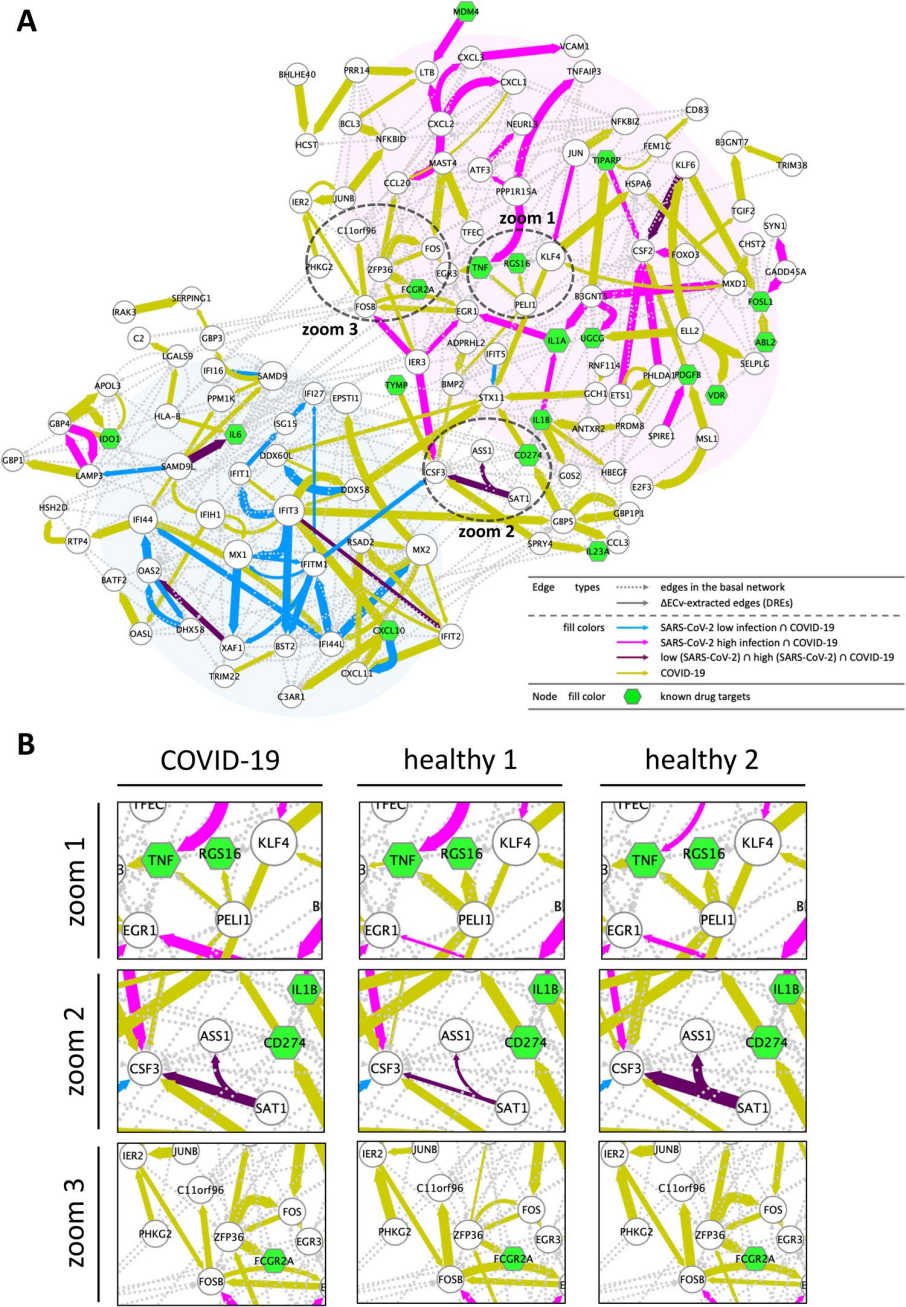
Establishment of the COVID-19 patient-specific individual network. (**A**) The COVID-19 patient-specific network with RCs represented by edge sizes. The network comprises 127 nodes and 412 edges (including 245 basal edges). The colored solid edges represent DREs; SARS-CoV-2 (high MOI: 2) $\cap$ COVID-19-perturbed (magenta), SARS-CoV-2 (low MOI: 0.2) $\cap$ COVID-19-perturbed (blue), SARS-CoV-2 (high MOI: 2) $\cap$ SARS-CoV-2 (low MOI: 0.2) $\cap$ COVID-19-perturbed (purple), COVID-19-perturbed exclusive edges (yellow). The dotted edges represent the basal edges (gray). The nodes (green) represents the known drug target genes (Supplementary Table S2). The node size stands for the extent of outdegree. (**B**) Zoomed regions indicated in Fig. 6A for three individuals (healthy 1, healthy 2, and the patient with COVID-19). RCs are represented as edge sizes to show individual differences.

To uncover the differences in the local regulatory system, we next examined the profiles of the COVID-19-perturbed network at an individual level using the RC method (Fig. 1). As the two COVID-19 samples were originally derived from a single patient who tested positive for COVID-19, we calculated RCs for three individuals (healthy 1, healthy 2, and COVID-19 patient). The depiction of the RC as the edge sizes eventually led to the establishment of the COVID-19 patient-specific network, which was likely to show how the cellular system changed in the patients with COVID-19 compared with the healthy controls (Fig. 6A). The panel of three individual networks dramatically exhibited a great magnitude of differences, showing that the cellular regulatory systems were quite distinctive among individuals (Supplementary Animation 2). As our approach captures differences in the system between COVID-19 and healthy individuals as a network, genes that are not normally considered to be up/down-regulated in healthy people will also be included in the network. In comparison with the SARS-CoV-2-perturbed network established by the well-organized *in vitro* experiments using cell lines (Supplementary Animation 1), this broad range of RC fluctuation for each *in vitro* sample likely reflects further differences among individuals. The representative regions where local regulatory systems are different among individuals are illustrated in Fig. 6B. In the zoom 1 region, PELI1 is a parent gene for both TNF and RGS16; these two signals were dominant in the healthy individuals, but not in the patient with COVID-19. In contrast, the zoom 2 and 3 regions showed that local signals were clearly different, not only between the healthy patients and the patient with COVID-19, but also even between two healthy individuals.

## Discussion

Here, we have presented the host cellular gene networks perturbed by SARS-CoV-2 both *in vitro* and *in vivo* by using our proposed framework for gene network analysis. As the networks we established to be associated with SARS-CoV-2 were generated through RNA-seq data, these networks explained how genes were systematically regulated at the transcriptome level. Although our approach depends on the initial network estimation with an experimental dataset and may therefore risk the inclusion of false relationships or the exclusion of true relationships, we have succeeded in capturing the biologically explainable immune response systems in human cells induced by SARS-CoV-2 at the level of signaling networks.

Sensing of viruses causes an immune defense system in host cells, which induces acute IFN signaling activation followed by the expression of IFNs. These IFNs amplify the JAK/STAT signaling to promote the expression of various ISGs and accelerate subsequent cytokine signaling^26^. As illustrated in Fig. 3, the mutually interacting module of ISGs (module 1) followed by the IFN and JAK/STAT signaling was shown to be an early response to SARS-CoV-2 infection. During the process of cells exposed to high SARS-CoV-2 viral loads, the signaling appears to move to the next stage, represented by inflammatory signaling, including the involvement of various cytokines (Fig. 3). The recently reported drug, dexamethasone, could be effective for patients with severe COVID-19 via suppression of these orchestrated inflammatory signaling cascades^28^. In this network (Fig. 3), IL6 was located as a hub gene to regulate downstream cascades, including chemokines and colony-stimulating factors, which have been reported to be increased in patients with COVID-19^21^. The web of chemokines, such as CXCL1, CXCL2, and CXCL3, may represent how the SARS-CoV-2-infected cells present a signal to induce leukocyte chemotaxis and infiltration. The localization of ICAM1 in the vicinity of IL6 and chemokines is supportive of this, as ICAM1 is known to be a scaffold for the accumulation of leukocytes at inflammatory sites and its expression is regulated by cytokines, including IL6^29,30^. This tendency was also observed in the network comparison analyses across the four respiratory viruses, including SARS-CoV-2 (Supplementary Fig. S1C). These data showed that IL6 was not exclusive to SARS-CoV-2, but a universal factor in response to respiratory viral infection, with the exception of the influenza A virus. Given that several studies have reported that tocilizumab, an inhibitor of the IL6 receptor, is a potential drug that can suppress the cytokine storm observed in many critical patients with COVID-19^31,32^, the accumulated evidence strongly suggests that IL6 is a central regulator of the inflammatory cascade, even from a network perspective. Additionally, our network showed that CSF2 was regulated by various factors, including IL6, which strengthened previous reports suggesting that CSF2 might be a promising therapeutic target in combination with IL6^33,34^. Moreover, other immune defense signaling pathways such as complement and macrophage were identified (Fig. 2C). In contrast, coagulation cascade reported abnormalities in patients with COVID-19^35^ was not identified, probably because this aberration of coagulation may have occurred at the physiological system level rather than at the cellular level. Our method only captures the system at the cellular level, and it is not yet possible to see the response of the entire biological system (e.g. between organs). This would be a limitation of our current approach.

Several recent studies have shown that ACE2 plays a key role in the process of SARS-CoV-2 infection. SARS-CoV-2 enters into host cells via ACE2^36^, and ACE2 was found to be an ISG in human airway epithelial cells^37^. Considering that the SARS-CoV-2-perturbed network includes several ISGs (Fig. 3), it can be reasoned that some clues regarding ACE2 may be present in this network. In this context, we found that ACE2 was closely located to this network and was downstream of TNFRSF9, ATF3, and ARRDC3 via ACHE (Supplementary Fig. S3); these are potential candidates for further investigation of the relationship between ACE2 and ISGs. Among them, ATF3 would be the most promising as it was found to be a direct transcriptional target for ACE2^38^. Thus, our networks provide promising information to elucidate SARS-CoV-2 profiles from a broad biological perspective.

Our second noteworthy outcome in this study was that we succeeded in the characterization of sample-specific individual networks by introducing the new edge-quantitative technique of RC. In particular, although it is impossible to estimate a network with a small number of samples, such as the four biopsy samples used in our case, the basal network model that was already obtained through the analysis of the *in vitro* dataset with both RC and ECv methods led to establishment of the COVID-19 patient-specific individual network. This process represents the method of extrapolation between *in vitro* and *in vivo* experiments. Each sample exhibits a unique regulatory profile, especially in actual individuals (such as those obtained from biopsy) rather than well-controlled *in vitro* samples (Supplementary Animation 1 and 2). These results probably reflect a more realistic clinical situation and increase the importance of the most effective utilization of a biopsy dataset. In the current outbreak of COVID-19, we need to look into both biological and clinical aspects to explore COVID-19 therapy. The individual networks regarding COVID-19 show the extent to which individuals possess their own network, which ultimately links to the necessity of a personalized treatment. Therefore, our efforts are a potential contribution to the emerging field of personalized medicine. The biopsy dataset that we used was not sufficient to allow interpretation of the comprehensive information through individual networks in patients with COVID-19, as it contained fewer COVID-19 samples. In addition, since the lung samples were obtained postmortem patients with COVID-19, we could not determine at what time point in disease progression the identified regulatory system is in this study (Fig. 6). More clinical samples with time series and disease severity information from patients with COVID-19 can lead to the determination of key regulatory systems at a clinical level. As data regarding SARS-CoV-2 has been currently accumulated by the efforts of researchers, we hope that our panel of network analyses will be of help to the SARS-CoV-2 research field and to establishment further treatments for COVID-19.

## Methods

### Global gene network estimation and core network extraction

In general, methods for gene network analysis are intended for the extraction of gene-to-gene regulatory relationships that universally underlie given transcriptome datasets. Unlike commonly existing gene networks, we recently developed a method to extract sample-specific gene networks. Our method first estimates a global gene network, called the *basal network*, which includes all the genes in a dataset using a Bayesian network with *B*-spline nonparametric regression^24,39^. Bayesian network estimation is capable of capturing global cause-and-effect relationships among gene expression, rather than extracting locally co-regulated genes, such as co-expression correlation networks. This is realized by finding the conditional independencies among the variables. In gene network analysis using a Bayesian network, gene expression is regarded as an observed sample from the random variables that correspond to genes or transcriptomes in a cell.

Let $X_1,\ldots ,X_p$ be *p* variables of genes. In a Bayesian network, we consider the joint density of *p* variables and assume that it is decomposed as the product of local conditional densities, such that

$$\begin{aligned} f(X_1,\ldots ,X_p; \theta _G) = \prod _{j=1}^{p} f(X_j|X_{j_{1}},\ldots ,X_{j_{q_j}}; \theta _j), \end{aligned}$$

where $j_1,\dots ,j_{q_j}$ are indices of $q_j$ dependent variables of the *j*-th variable. This decomposition can be represented as a directed acyclic graph (network), and variables $X_{j_1}, \ldots , X_{j_{q_j}}$ are connected as parents or inputs of the *j*-th variable in the network.

The *B*-spline nonparametric regression version of the Bayesian network models gene-to-gene expression relationships as mathematical equations using *B*-spline curves, such that

1 $$\begin{aligned} x_j = m^{(j)}_{1}(x_{j_1}) + \cdots + m^{(j)}_{q_j}(x_{j_{q_j}}) + \varepsilon _j, \end{aligned}$$

where $x_j$ represents the expression value of the *j*-th gene, $\varepsilon _j$ is the error term normally distributed with mean 0 and variance $\sigma _j$, and $m^{(j)}_{k}(x) = \sum _{m=1}^{M} \gamma _{jkm} b_{jkm}(x)$ is a regression function using *M* *B*-spline basis functions $b_{jkm}(\cdot )$, and their coefficients $\gamma _{jkm}$.

The structure search of the Bayesian network corresponds to finding the decomposition of the joint density. This is implemented by maximizing the posterior probability such that

$$\begin{aligned} p(G|X) \propto \pi (G) \int \prod _{i=1}^{n} f(x_{i1},\ldots ,x_{ip}; \theta _G) \pi (\theta _G|\lambda ) d \theta _G, \end{aligned}$$

where *X* is an *n*-by-*p* input matrix whose element $x_{ij}$ corresponds to an expression value of the *i*-th sample for the *j*-th gene, *G* represents the network structure, $\pi (G)$ is the prior probability of *G*, $\theta _G$ is the parameter vector of the local conditional densities, $\pi (\theta _G|\lambda )$ is the prior distribution of $\theta _G$, and $\lambda$ is the hyperparameter vector. The difficulty in gene network estimation by the Bayesian network is the step involving structure learning for large networks, as this is known to be an NP-hard problem, in other words, an exponential increase in search space due to the number of variables. We used the neighbor node sampling and repeat algorithm that realizes the estimation of the large Bayesian network structure^24^. It repeats the subnetwork estimation many times in parallel for the sampled variable sets by random walking, and thus it can estimate a large network within a realistic time.

After the basal network estimation, we then quantified every single edge with respect to a certain sample in terms of the system-level usage of the edge with the estimated mathematical model. Tanaka et al.^14^ defined an *edge contribution value* (ECv) of edge $j_k \rightarrow j$ as $ECv_{(u)}(j_k \rightarrow j) = m^{(j)}_{k}(x^{(u)}_{j_k})$ where $x^{(u)}_{j_k}$ represents the expression value of the $j_k$-th gene in a certain sample denoted by *u*, and $m^{(j)}_{k}(\cdot )$ is a regression function defined in Eq. (1). Note that sample *u* did not necessarily have to be a single sample for use in the network estimation. They proved that ECv could be used for the quantification of edge $j_k \rightarrow j$ with respect to a given sample. To extract sample-specific networks, they considered the differences of ECvs between two different conditions of samples, similar to extracting differentially expressed genes by comparing control and perturbed expressions. They defined $\Delta$ECv as

2 $$\begin{aligned} \Delta ECv_{(S,T)}(j_{k}\rightarrow j) = \left| \frac{1}{|S|} \sum _{s \in S} ECv_{(s)}(j_{k}\rightarrow j) - \frac{1}{|T|} \sum _{t \in T} ECv_{(t)}(j_{k}\rightarrow j) \right| , \end{aligned}$$

where *S* and *T* are sets of samples observed in the particular conditions, respectively, in which $|S| \ge 1$ and $|T| \ge 1$. Note that in the case of $|S|=|T|=1$, this allows consideration of the differences between just two samples, for example, control and perturbed samples. In general, we assume multiple replicated samples or a set of individual samples for both *S* and *T*. By extracting edges and their connected nodes with $\Delta$ECvs greater or equal to a certain threshold, we can define the sample- or condition-specific core network from the basal network. In this study, *S* and *T* were sets of infected and control (mock) replicated samples, respectively. As the target dataset includes control samples for a particular series of experiments, we can extract certain core networks from them by calculating $\Delta$ECv for the series of experiments using their corresponding control samples. For example, we extracted a SARS-CoV-2-perturbed core network by calculating $\Delta$ECvs for the SARS-CoV-2-infected and their corresponding mock-triplicate samples. As performed in the previous study^14^, we generally employ $\Delta \text {ECv} \ge 1.0$ for the threshold and carry out statistical t-tests to extract a core network. This threshold approximately corresponds to 2-fold changes in differentially expressed genes for the extracted genes. Thus, we considered that the extracted networks, including edges and nodes, were significantly activated by the infection in cellular regulatory systems. In this study, we did not carry out statistical tests due to the small number of samples.

### Proposed relative contribution of edges for characterization of individual networks

This ECv development allowed for a new solution for gene network analyses. In the previous study^14^, we succeeded in characterizing network profiles by calculating ECvs for edges in a $\Delta$ECv-extracted core network with respect to many samples from patients with cancer. Conventional clustering onto these calculated ECvs led to the identification of prognosis-related subgroups. Thus, we demonstrated that the differences and similarities in the edge profiles of the network could be captured as patterns of ECvs. Despite the high availability of ECvs, it is impossible to directly compare ECvs between individual samples because ECvs have different sizes depending on the estimated pairwise edge and the sample. The normalization of ECvs across samples is inappropriate for our purpose due to the mutual dependency of the individual network on each sample. Thus, it is not possible to highlight the differences in regulatory systems at an individual level.

For these reasons, ECvs are not appropriate for the analysis of individual networks. To overcome these drawbacks, we have proposed a novel method, *relative contribution* (RC), to quantify edges with respect to individual samples using the estimated gene network model. We hypothesized that the differences in individual samples in terms of the cellular system could be attributed to the differences in the ratios of the contributions of edges connecting to a certain node in the network. Edges with different samples need to be described as differently weighted edges according to the ratios of effects between parents that regulate or are connected to a certain gene. Additionally, the quantification of a network with a single sample needs to be independent from other samples and their distributions. To achieve this, we define the relative contribution of an edge with respect to a sample as

$$\begin{aligned} RC_{(u)}(j_k \rightarrow j) = \frac{|ECv_{(u)}(j_k \rightarrow j)|}{\max _{1 \le k\prime \le q_j} |ECv_{(u)}(j_{k\prime } \rightarrow j)|}, \end{aligned}$$

where *u* represents a certain sample ($0 < RC \le 1$). That is, an RC of the edge is the relative strength of the contribution of the edge to the maximum strength among the parents connecting to the same child node. The reason why an RC is not divided by the sum of the ECvs is that the range of RCs does not shrink depending on the number of parents of the child node. One drawback of RCs is that if the ratio of ECvs of the parents is not changed, the changes in parent values do not affect the RCs. However, the RCs of their downstream edges will be affected by such changes. Therefore, this drawback is not problematic in terms of the specification of differences in individual networks. Note that, similar to ECvs, sample *u* does not necessarily need to be a single sample used for the network estimation. As illustrated in the Results section, we have shown that RCs can be used to analyze individual networks, even if we have a single sample, or only a few samples, of gene expression data, as long as a basal network can be estimated from other datasets. RC, therefore, offers a significant enhancement to our framework for gene network analysis. Our data have demonstrated that the framework, through an integration of the three key pieces—Bayesian network estimation, ECv, and RC—provides a powerful data-driven solution to seek biological phenomena through cellular systems ranging from a global level to an individual level. Our proposed framework is mathematically illustrated in Supplementary Fig. S4.

### Dataset

The transcriptome dataset GSE147507 was downloaded from the NCBI Gene Expression Omnibus^7^. The samples were infected with respiratory viruses, including SARS-CoV-2, and biological replicates were performed. We first selected samples exclusive for human RNA-seq with 78 samples. The detailed descriptions of samples are listed in Supplementary Table S1, which was created according to the source paper^7^. Among the samples, four samples of the *in vivo* experiment (biopsy) data were pre-eliminated. The $\log _2$-transformed dataset was filtered to remove genes with a mean percentile lower than 30%, resulting in 74 samples and 15,258 genes. This preprocessed dataset of the 74 $\times$ 15,258 matrix was used as input for the basal network estimation. The biopsy dataset eliminated above, prior to global network estimation, consisted of four samples (two healthy samples and two COVID-19-positive samples). The RPM (reads per million)-normalized biopsy dataset was $\log _2$-transformed, and genes with at least one zero value were removed to obtain more reliable data. The two technical replicate samples for COVID-19 were averaged for the RC calculation. Following this preprocessing, the input dataset for the RC calculation finally comprised a 3 $\times$ 4,516 matrix. The RNA-seq samples used for $\Delta$ECv calculations in this study were: SARS-CoV-2 in A549 cells (MOI of 0.2/2 for 24 hr, n=3) and the corresponding mock (n=3); SARS-CoV-2 in normal human bronchial epithelial (NHBE) cells (MOI of 2 for 24 hr, n=3) and the corresponding mock (n=3); SARS-CoV-2 in Calu-3 cells (MOI of 2 for 24 hr, n=3) and the corresponding mock (n=3); human respiratory syncytial virus (RSV) in A549 cells (MOI of 2 for 24 hr, n=3) and the corresponding mock (n=3); human parainfluenza virus 3 (HPIV3) in A549 cells (MOI of 2 for 24 hr, n=3) and the corresponding mock (n=3); influenza A virus (IAV) in A549 cells (MOI of 5 for 9 hr, n=2) and the corresponding mock (n=2); COVID-19 (n=2) and healthy (n=2).

### Pathway analysis

The canonical pathway analysis was performed through the use of Ingenuity Pathway Analysis software^40^.

### Network analysis and visualization

The network visualization and the network analysis were performed using Cytoscape (version 3.7.2 and 3.8.0)^41^. The genes for known drug targets were acquired from IPA knowledge database^40^ and the representative drugs were listed in Supplementary Table S2.

### Computational environments

All the computations for the network estimation and the ECv calculations in this study were performed by the SHIROKANE supercomputer system (Shirokane5) at Human Genome Center, the Institute of Medical Science, the University of Tokyo, where the computation nodes were equipped with dual Intel Xeon Gold 6154 3.0GHz CPUs and 192GB memory per node.

## Supplementary Information

Supplementary Information 1.

Supplementary Information 2.

Supplementary Information 3.

Supplementary Information 4.

Supplementary Information 5.

Supplementary Information 6.

Supplementary Information 7.

Supplementary Information 8.

Supplementary Information 9.

Supplementary Information 10.

## Data Availability

All the network files generated in this study are provided in the supplementary data. The networks are available at NDEx (see Supplementary Information). The program for network estimation is freely available for SHIROKANE users. The ECv/RC calculation program is available for non-commercial academic users from the corresponding author upon request.
