# Supplementary figures and images for: Dynamic changes in gene-to-gene regulatory networks in response to SARS-CoV-2 infection
Source: Sci Rep. 2021 May 27;11:11241. doi: 10.1038/s41598-021-90556-1 (PMC8160150; doi:10.1038/s41598-021-90556-1)

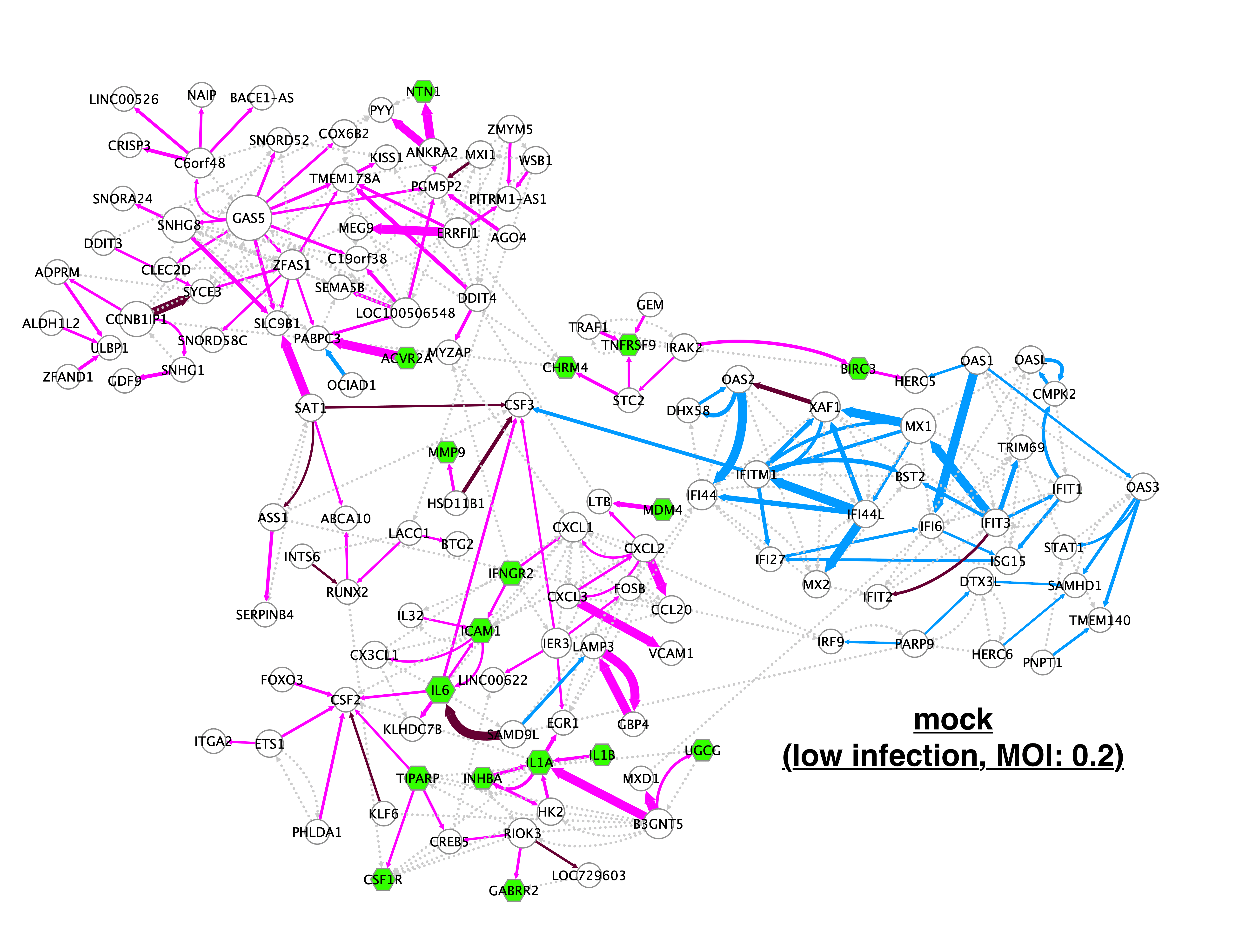

Supplement: Supplementary file 6 — Supplementary Information 6. [file 41598_2021_90556_MOESM6_ESM.gif]

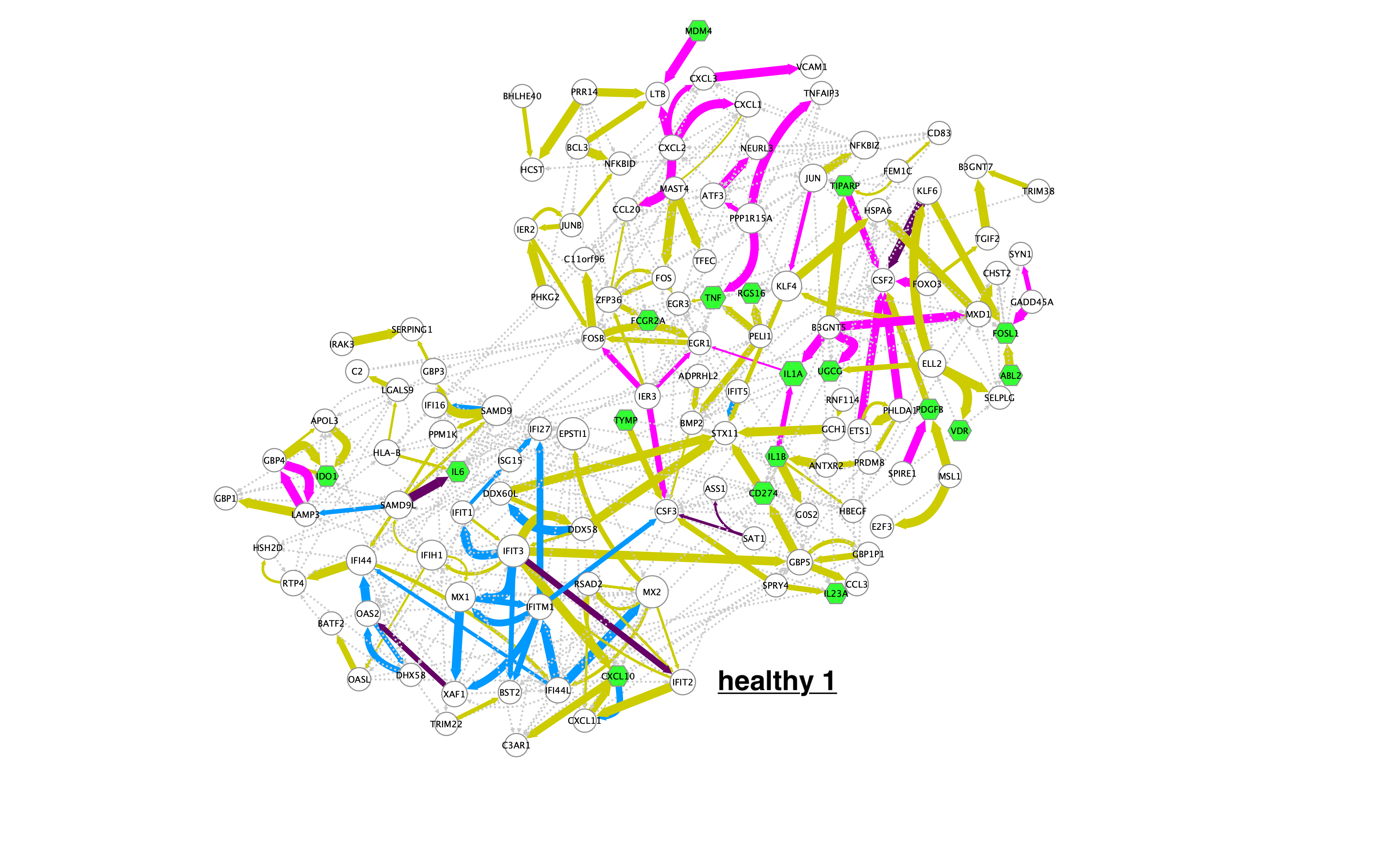

Supplement: Supplementary file 7 — Supplementary Information 7. [file 41598_2021_90556_MOESM7_ESM.gif]
