# Supplementary material for: Dynamic changes in gene-to-gene regulatory networks in response to SARS-CoV-2 infection
Source: Sci Rep. 2021 May 27;11:11241. doi: 10.1038/s41598-021-90556-1 (PMC8160150; doi:10.1038/s41598-021-90556-1)
Supplement: Supplementary file 10 — Supplementary Information 10. [file 41598_2021_90556_MOESM10_ESM.pdf]

Supplementary Information

Dynamic change in gene-to-gene regulatory network in response to SARS-CoV-2

Authors:
Yoshihisa Tanaka, Kako Higashihara, Mai Adachi Nakazawa, Fumiyoshi Yamashita, Yoshinori Tamada, and Yasushi Okuno

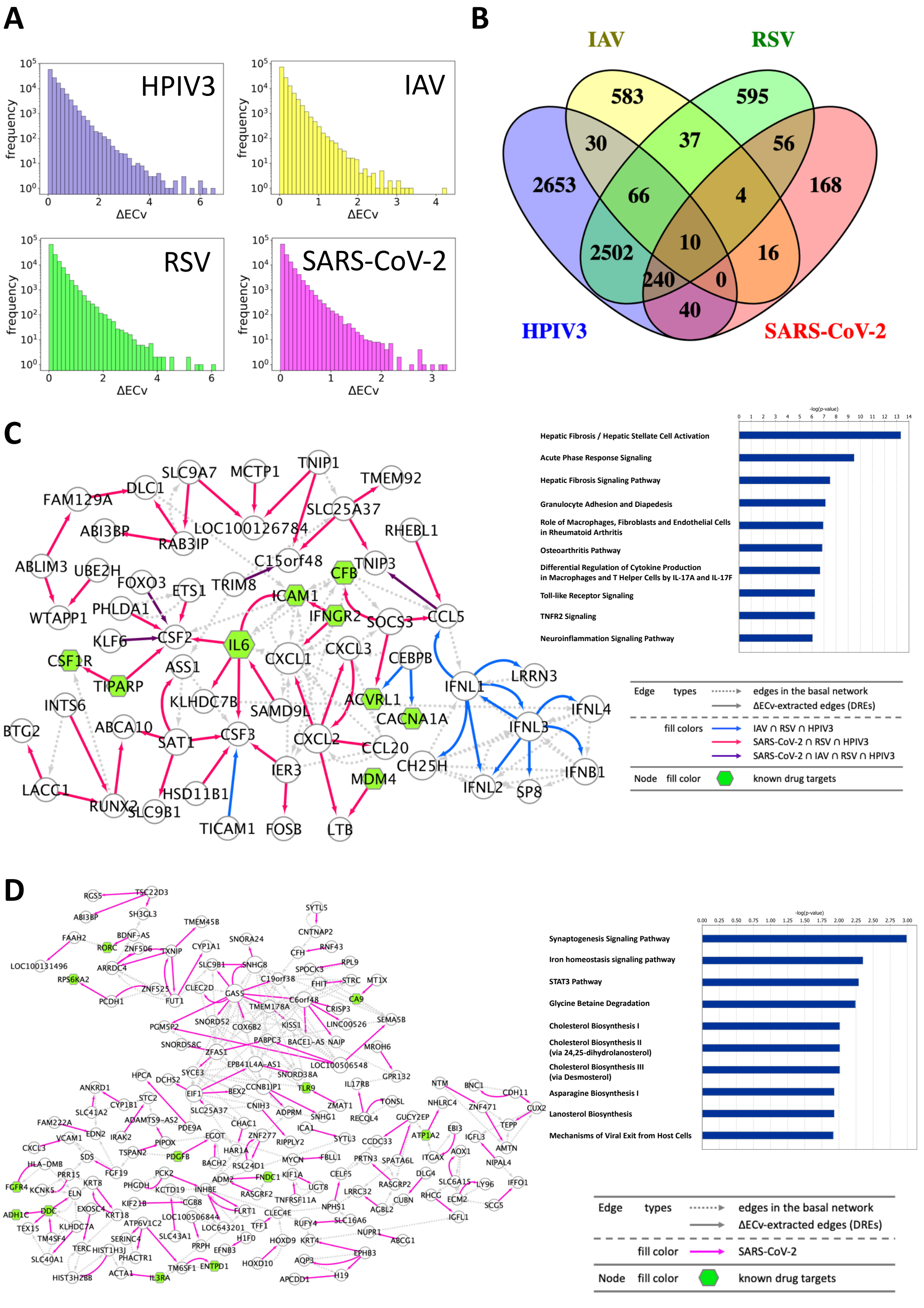

Supplementary Figure S1: Network comparison analyses across four respiratory viruses. Comparisons between these viruses were performed under the same conditions as the original paper. (**A**) The histograms of ΔECv for each respiratory virus as indicated: SARS-CoV-2 (MOI: 2), HPIV3, IAV, and RSV. ΔECvs were calculated following Eq. (2) where S = virus-infected and T = corresponding mock samples for each virus (see Methods). The X-axis corresponds to the threshold for each ΔECv. The Y-axis stands for the number of edges on a log scale. (**B**) The Venn diagram represents the numbers of ΔECv-extracted edges for all respiratory viruses with a ΔECv threshold of 1.0. (**C**) The respiratory viruses-shared network comprised 62 nodes and 116 edges (including 53 basal edges). The colored solid edges represent DREs; SARS-CoV-2 ∩ IAV ∩ HPIV3 ∩ RSV (purple), SARS-CoV-2 ∩ RSV ∩ HPIV3 (red), IAV ∩ RSV ∩ HPIV3 (blue). The top 10 terms of canonical pathway analysis for the genes of ΔECv-extracted DREs shared by at least three viruses in the Venn diagram (Supplementary Fig. S1B). (**D**) The SARS-CoV-2 specific network comprising 182 nodes and 295 edges (including 171 basal edges). The solid edges (magenta) represent DREs for SARS-CoV-2 (MOI: 2). The dotted edges represent the basal edges (gray). The size of the node represents the extent of outdegree. The nodes (green) are target genes for existing drugs (Supplementary Table S2). The top 10 terms of canonical pathway analysis for the genes of ΔECv-extracted DREs exclusive for the SARS-CoV-2 in the Venn diagram (Supplementary Fig. S1B).

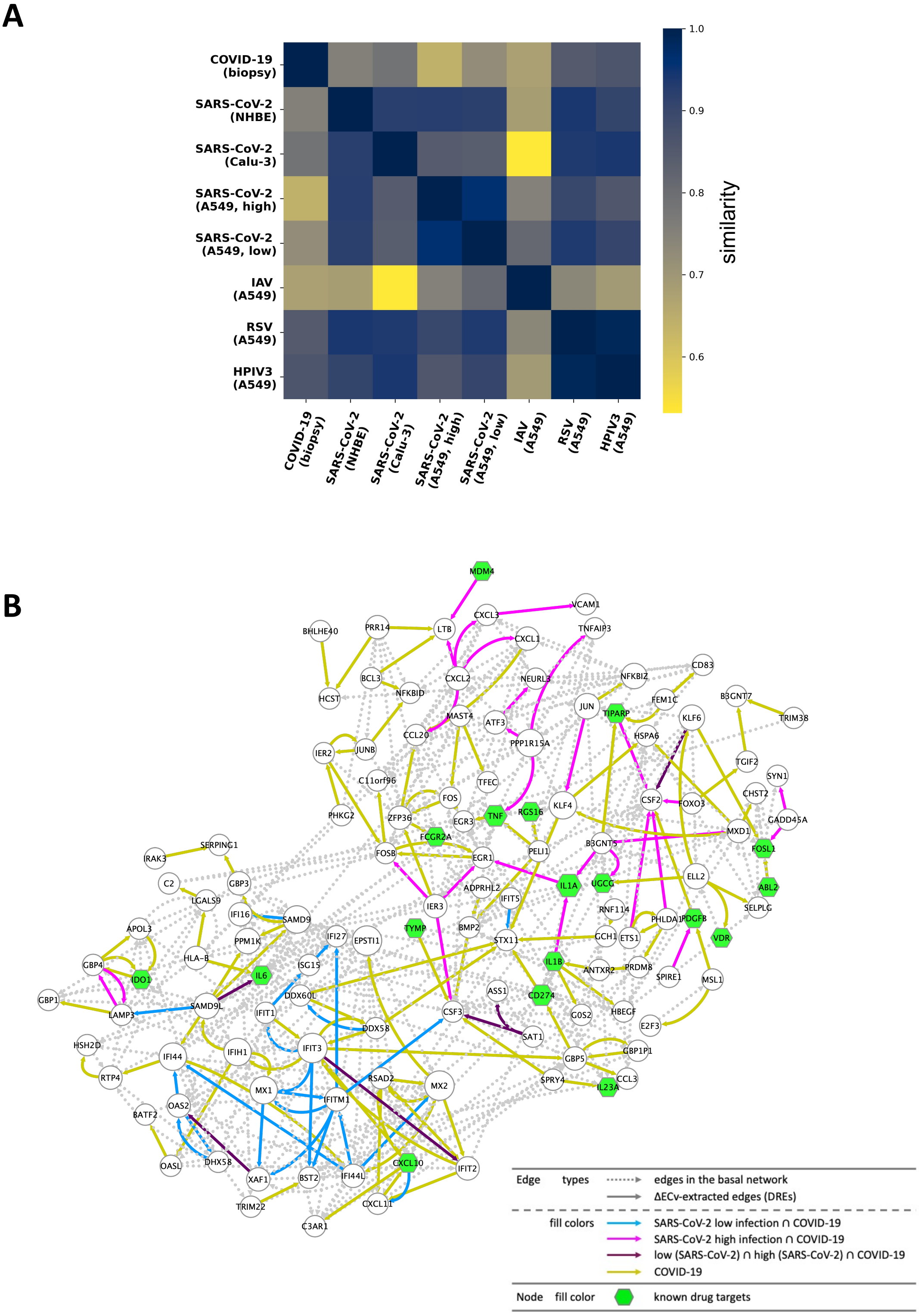

Supplementary Figure S2: Multiple analyses for generating the COVID-19-perturbed network. (**A**) The similarity heatmap is shown and samples for comparisons are labeled as indicated. Similarity is calculated with cosine distance method for ECvs of the 167 DREs. (**B**) The COVID-19 patient-specific network in combination with the Venn diagram analysis (Fig. 5D). The network is composed of 127 nodes and 412 edges (including 245 basal edges). The colored solid edges represent DREs; SARS-CoV-2 (high MOI: 2) ∩ COVID-19-perturbed (magenta), SARS-CoV-2 (low MOI: 0.2) ∩ COVID-19-perturbed (blue), SARS-CoV-2 (high MOI: 2) ∩ SARS-CoV-2 (low MOI: 0.2) ∩ COVID-19-perturbed (purple), COVID-19-perturbed exclusive edges (yellow). The dotted edges represent the basal edges (gray). The nodes (green) represent the known drug target genes (Supplementary Table S2). The node size represents the extent of outdegree.

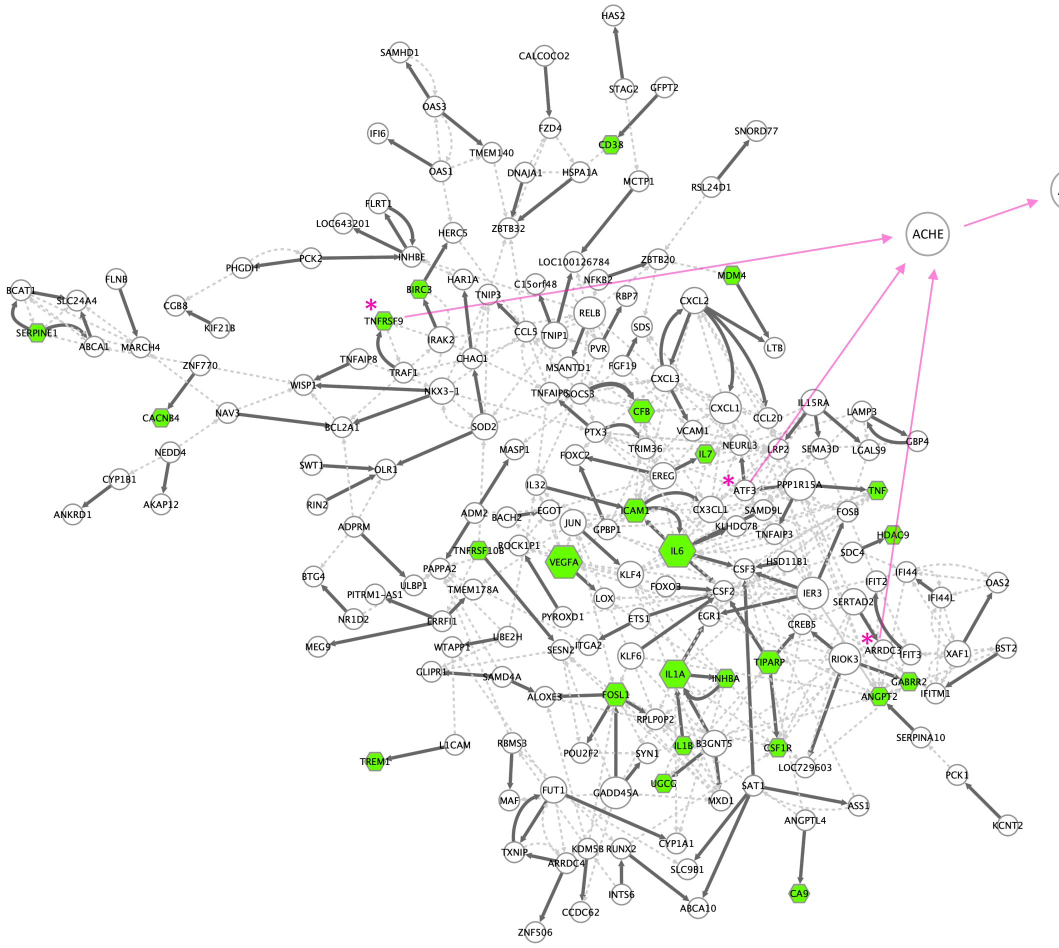

Supplementary Figure S3: The schematic network illustration of ACE2 involvement in immune defense system of host cells. The network (188 nodes and 420 edges) was generated by the ΔECv-extracted edges (black) shared with A549, NHBE and Calu-3 cells in response to SARS-CoV-2 infection. The nodes (green) represent the known drug target genes. The edges (magenta), and nodes (marked magenta) represent potential regulatory signalings to ACE2 via ACHE. The dotted edges represent the basal edges (gray).

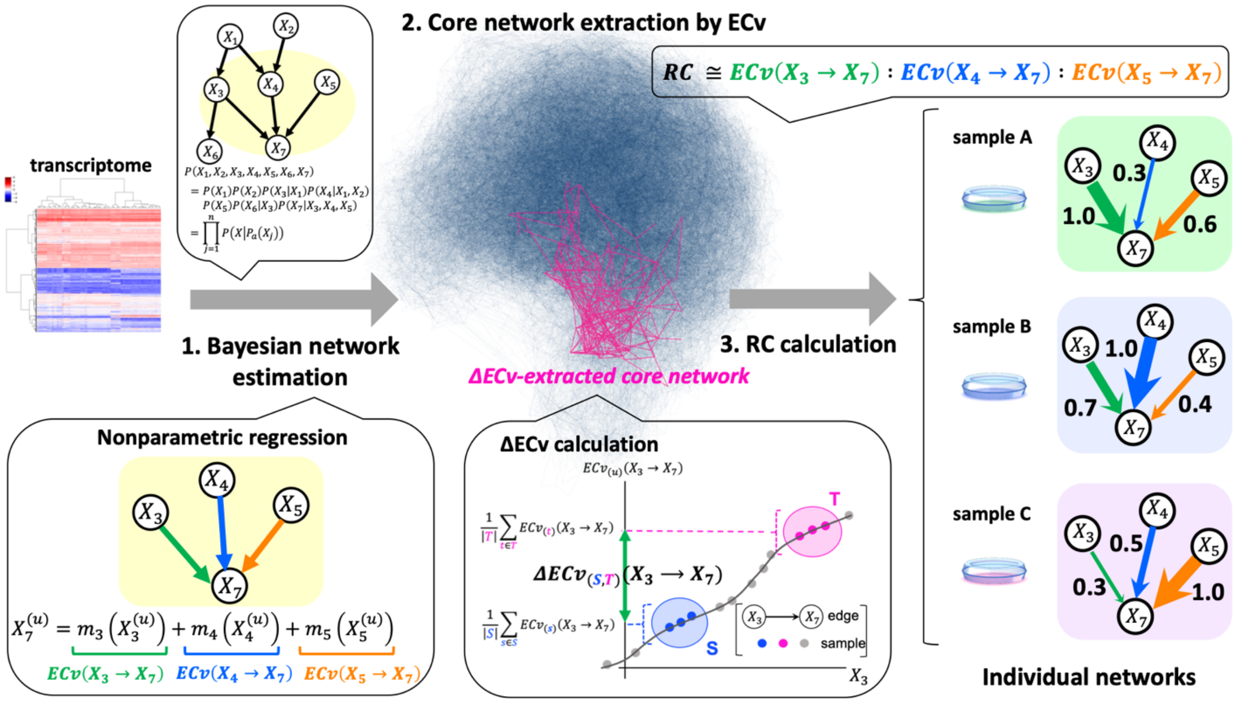

Supplementary Figure S4: Mathematical illustration of our proposed framework for the gene network analysis. The centered hairball (blue) represents a basal network. The network (magenta) is a core network extracted by the ΔECv calculation.

Supplementary Files

ECvmatrix_vitro.zip: ECv matrix for in vitro dataset
ECvmatrix_biopsy.zip: ECv matrix for biopsy dataset
RCmatrix_vitro.zip: RC matrix for in vitro dataset
RCmatrix_biopsy.zip: RC matrix for biopsy dataset
basalnetwork.zip: basal network file

Networks at NDEx

The networks used in this study are available at NDEx (http://www.ndexbio.org/#/networkset/ebf22964-f1b2-11ea-99da-0ac135e8bacf).

- The basal gene network in the involvement of respiratory viruses infection including SARS-CoV-2. (http://www.ndexbio.org/viewer/networks/116f99ca-f0fd-11ea-99da-0ac135e8bacf)

- The SARS-CoV-2-perturbed network. (http://www.ndexbio.org/viewer/networks/25708de8-f1b1-11ea-99da-0ac135e8bacf)

- The COVID-19 patient biopsy (basal) network. (http://www.ndexbio.org/viewer/networks/5ab94147-f0f7-11ea-99da-0ac135e8bacf)

- The COVID-19 patient-specific individual network. (http://www.ndexbio.org/viewer/networks/e6e86f53-f1ad-11ea-99da-0ac135e8bacf)

Supplementary Animations

Supplementary Animation 1: Sample-specific individual networks in the SARS-CoV-2-perturbed network.
The RC-introduced individual networks are shown with animation for the representative four samples from each group (mock for SARS-CoV-2-infection (low MOI: 0.2), SARS-CoV-2-infection (low MOI: 0.2), mock for SARS-CoV-2-infection (high MOI: 2), SARS-CoV-2-infection (high MOI: 2)). The depicted network is established in Fig. 3 (the SARS-CoV-2-perturbed network). The region of module 3 is shown in Fig. 4. RCs are represented as edge sizes to show individual differences.

Supplementary Animation 2: Individual network in the COVID-19-perturbed network.
The RC-introduced individual networks are shown with animation for three individuals (healthy 1, healthy 2, and patient with COVID-19). The depicted network is established in Fig. 5 and 6 (the COVID-19-perturbed network). RCs are represented as edge sizes to show individual differences.

Supplementary Tables

Supplementary Table S1: The detailed list of sample description.
Supplementary Table S2: The gene list for known drug targets.
